# Verbal Abuse in the Operating Room: A Survey of Three General Hospitals in the Peloponnese Region

**DOI:** 10.7759/cureus.18098

**Published:** 2021-09-19

**Authors:** Maria Saridi, Aikaterini Toska, Dimitra Latsou, Anastasia Giannakouli, Mary Geitona

**Affiliations:** 1 Epidemiology and Public Health, General Department of Lamia, University of Thessaly, Lamia, GRC; 2 Social and Educational Policy, University of Peloponnese, Corinth, GRC

**Keywords:** verbal abuse, victim, operating room, nurses, physicians

## Abstract

Background

The operating room is a special place in a hospital structure, which has a very high psychological load and many moments of tension, often leading to difficulties in communication between the health professionals involved, as well as cases of verbal and physical violence.

Purpose

The purpose of the study was to investigate the existence of verbal violence in the operating rooms of three general hospitals in the Peloponnese region of Greece.

Methods

A cross-sectional survey was conducted of health professionals (physicians and nurses) employed in the operating rooms of three general hospitals in the Peloponnese region. For the data collection, the Verbal Abuse Scale questionnaire (VAS) was used. A total number of 80 questionnaires were self-completed and collected. The statistical analysis was performed with the SPSS Statistics software v.25 (IBM Corp., Armonk, NY).

Results

Regarding the frequency of verbal violence faced by health professionals, 36.8% of physicians stated that they experience verbal violence once a year whereas more than 20% of nurses reported that such incidents occur more than once a month (p=0.148). For the physicians, the abuser was usually their supervisor, while for the nurses, a physician. The majority of physicians stated that they felt mainly anger (2.94 ± 1.35), disgust (2.58 ± 1.54), and sadness/hurt (2.35 ± 1.37) after the verbal abuse, whereas most of the nurses felt anger (3.49 ± 1.39), disgust (3.05 ± 1.52) and frustration (2.95 ± 1.47).

Conclusion

Nurses are more often the victims of verbal abuse than physicians and are more likely to feel frustrated after experiencing verbal abuse. Gradual change to the organizational culture is an important measure to stop the occurrence of such incidents.

## Introduction

Workplace violence is a term used to describe abusive behavior that is directed at others with the intent to cause harm in the setting of the work environment. The increasing frequency of violence in the work environment is a major concern for employees and employers, recognized by the World Health Organization (WHO) as a key health priority [1]. According to WHO, violence at work is defined as the result of complex multi-stakeholder interactions, with an emphasis on working conditions and organization [2].

The harmful effects of violence can be difficult to recognize, especially when they have no physical impact. Studies have revealed the negative effects on employees' mental and physical health, which in the latter case can manifest in the form of symptoms such as pain and palpitations [3,4]. The effect of this situation on employees' efficiency is also a given and can often lead to abandonment of the profession. The frequency of exposure to violence in the workplace seems to increase the incidence of health problems, which often turn serious [5-7].

International studies confirm findings of frequent exposure to violence in the workplace of health professionals, whether physical or psychological [8-10]. Psychological violence has been shown to be more prevalent than physical violence, reaching an average of 2.29 episodes of verbal aggression per eight hours of work [10,11]. Studies have shown that nurses are more exposed to violence than other health professional categories [12-14]. It is estimated that internationally, one in three nurses has been subjected to violence in the workplace mainly by patients and relatives, whereas nurses working in public hospitals appear to be at greater risk of violence in the workplace than those working in private hospitals [2,12]. In 2014, more than 9,000 healthcare professionals experienced work-related violence injuries that resulted in a few days off work. To address this issue, the US National Institute for Occupational Safety and Health (NIOSH) developed an award-winning online workplace violence prevention course for nurses in 2017 [13-15].

Verbal violence is a common form of violence in hospitals, leading to aggravating effects on the psychology of health professionals, and is a significant contributor to burnout [16]. A systemic review of 136 studies revealed that verbal abuse episodes occurred in more than 50% of violence cases at a global level. While the lowest rate is in Asia, it was almost twice as widespread in the Middle East [17].

Due to the special nature of the operating room in the hospital structure, it has a very high psychological load and the most moments of tension, which can often cause difficulties in communication between health professionals involved, as well as cases of verbal and physical violence. Multiple factors have been identified as causes for the rapid spread of verbal violence in the operating room [2,13]. A study by Flin et al. (2003) reported that 91.1% of the 1,000 operating room nurses surveyed had experienced verbal violence during their careers [18].

It is clear, however, that verbal violence is a peculiar situation, which is difficult to be clarified even if the confusing factors that can complicate this situation are removed. The purpose of the study is to investigate the existence of verbal violence in the operating rooms in three general hospitals of the Peloponnese region.

## Materials and methods

Methods

In order to achieve the aim of the study, a survey was conducted with health professionals (physicians and nurses) working in the operating room at three general hospitals in the Peloponnese region. According to data of the selected hospitals, 104 health professionals were employed. From the 104 questionnaires that were given, 80 were returned fully completed, resulting in a response rate of 76.9%. The rest (24 health professionals) answered that they did not experience verbal abuse, so they were excluded from the study. The collection of the questionnaires was carried out by the method of self-completion by the operating room health professionals. The duration of the survey was from August 2018 to January 2019.

Study questionnaire

The research tool consisted of two sections. In the first section, sociodemographic data of the employees were recorded. In the second section, verbal violence in the workplace was assessed, using the Verbal Abuse Scale (VAS) questionnaire, which has been translated and validated into Greek by Malliarou et al. (2015), used for the present study with the authors' permission [19]. The questionnaire consisted of 11 groups of questions, such as the frequency of the verbal abuse, the working relationship with the abuser, if other persons were present, and the potential work stress from the verbal violence. The remaining group of questions was related to the frequency and severity of 10 different examples of verbal abuse. The majority of questions were scored on a 5-point Likert scale where 1 represents "not at all" and 5 "very much". Other questions were scored on 7-point Likert scales, with one set where 1 represents "never" and 7 "every day" and another set where 1 represents "not at all" and 7 "extremely."

Ethics

Relevant permission to carry out the study was requested and obtained by all three hospital administrations involved (Ethics Committee of General Hospital [GH] of Corinth, Protocol no. 30500 30/07/2018; Ethics Committee of GH of Argos, Protocol no. 33483 30/11/2018; Ethics Committee of GH of Nauplio, Protocol no. 8281 21/12/2018). Anonymity and confidentiality were maintained in all stages of the survey; moreover, the participants provided informed consent to their participation in the study.

Statistical analysis

The processing of data was based on the presentation of descriptive statistics such as percentages, means, and standard deviations. The variables followed a normal distribution and so the parametric tests were selected. Pearson's chi-squared test and contingency tables were used for quality variables, while for quantitative variables compared to binary variables, Student’s t-test was performed. The level of statistical significance was set at 0.05. The program SPSS Statistics v. 25 was used for data analysis (IBM Corp., Armonk, NY).

## Results

Socio-demographic and occupational characteristics

The majority of physicians were men (72.5%), while the majority of the nurses were women (77.5%). Around 56% of physicians and 51.4% of nurses belonged to the age group 45 years and up. Table 1 presents the sociodemographic and occupational characteristics of the surveyed health professionals.

**Table 1:** Sociodemographic and occupational characteristics

Sample characteristics	Physicians		Nurses	
	No.	%	No.	%
Gender				
Male	29	72.5	9	22.5
Female	11	27.5	31	77.5
Age group				
<45 years	18	44.0	18	48.6
>45 years	22	56.0	19	51.4
Postgraduate degrees				
No	19	47.5	36	90.0
Masters	11	27.5	4	10.0
PhD	10	25.0	0	0
Years of experience in hospital				
<15 years	13	32.9	7	17.5
>15 years	27	67.1	33	82.5

Frequency of verbal abuse

**Figure 1:**
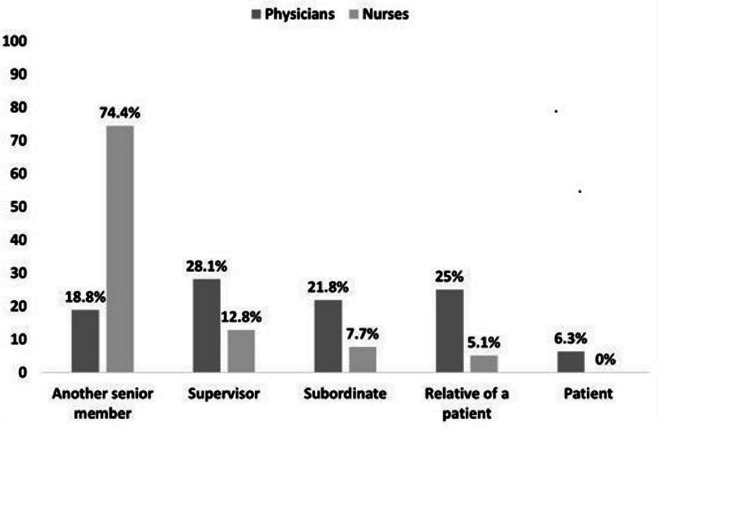
Working relationship between the victim of verbal abuse and the abuser

Around 79.4% of physicians stated that the episode of verbal abuse took place in the presence of other persons, in contrast to the nursing staff at 91.7% (p=0.143). Regarding the working relationship between the victim of verbal abuse and the abuser, 28.1% of the physicians answered that the abuser was their supervisor, while 74.4% of nurses stated that their abuser was usually another senior member, and in many cases, specifically a physician (Figure 1).

The results on the frequency of verbal abuse among health professionals in recent years are notable (Table 2). In particular, 38.9% of physicians stated that nurses have been subjected to violent verbal behavior more than once a year; however, 32.5% of nurses estimate that such incidents occur once a month (p=0.006). Regarding the frequency of verbal violence faced by health professionals, 36.8% of physicians stated that they have received verbal violence once a year, whereas more than 20% of nurses answered that they experienced such incidents more, often once a month (p=0.148) (Table 2).

**Table 2:** Frequency of verbal abuse

Verbal abuse occurrence	Nurses’ frequency of verbal abuse					Frequency of verbal abuse					
	Physicians			Nurses		Physicians			Nurses		
	No.		%	No.	%	No.	%	No.		%	
Never	1	2.8		0	0.0	5	13.2	2		5.0	
Once a year	1	2.8		0	0.0	14	36.8	10		25.0	
Several times a year	14	38.9		3	7.5	12	31.6	9		22.5	
Once a month	7	19.4		6	15.0	1	2.6	8		20.0	
Once a week	5	13.9		10	25.0	3	7.9	5		12.5	
Several times a week	7	19.4		13	32.5	2	5.3	3		7.5	
Every day	1	2.8		8	20.0	1	2.6	3		7.5	

Levels of stress resulting from violent verbal behavior

**Figure 2:**
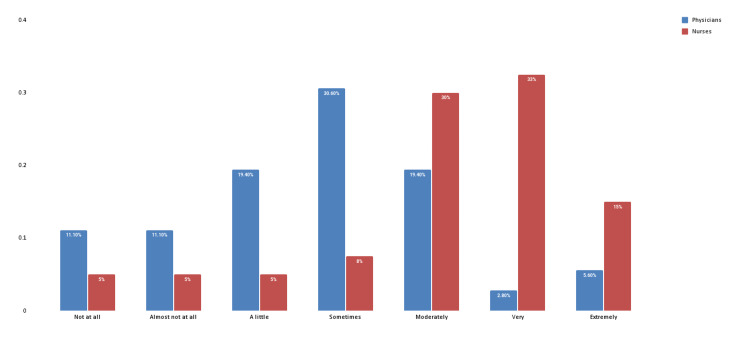
Levels of violent verbal behavior

In total, 55.6% of the physicians stated that the abuser didn’t understand the result of their abusive behavior, while 70.3% of the nurses stated that the abuser knew the consequences of their actions (p = 0.026). Also, for the 30.6% of the physicians, the abusive incident was sometimes stressful. In contrast, the majority of nurses (78%) highlighted that the event was moderately, very, or extremely stressful (p≤ 0.001) (Figure 2).

Frequency and stressfulness of verbal abuse forms

The subscale frequency and stressfulness of verbal abuse of the questionnaire record the frequency and severity of 10 different cases of verbal violence (Table 3). Ignoring (2.77±1.38) and accusing/blaming (2.73±1.31) were cited by the sample of physicians as the most common forms of verbal abuse that they experienced while judging/criticizing (3.32±1.31) and accusing/blaming (3.08±1.52) were reported as the most stressful action. The results for the sample of nurses were different since accusing/blaming (3.29±1.37) and judging/criticizing (3.32±1.35) were reported as the most common and the most stressful verbal abuse forms experienced. Additionally, a statistically significant difference was found between the two health professionals’ categories as far as the frequency and stressfulness of discounting. However, nurses were more likely to experience discounting compared to physicians, which led to higher levels of stress (p= 0.023 and p= 0.039, respectively).

**Table 3:** Frequency and stressfulness of verbal abuse forms between physicians and nurses

Frequency and stressfulness of verbal abuse	Frequency		P-value	Stressfulness		P-value
Victims’ Responses to Verbal Abuse	Physicians	Nurses		Physicians	Nurses	
Abusive anger	2.56±1.40	2.55±1.28	0.977	2.93±1.30	2.59±1.32	0.337
Condescending	2.24±1.25	2.50±1.30	0.526	2.18±1.24	2.17±1.25	0.982
Abuse disguised as a joke	2.65±1.27	3.13±1.43	0.208	2.30±1.26	2.92±1.41	0.114
Ignoring	2.77±1.38	2.46±1.36	0.437	2.68±1.46	2.38±1.36	0.489
Trivializing	2.33±1.28	2.90±1.37	0.142	2.90±1.51	3.23±1.50	0.465
Accusing and blaming	2.73±1.31	3.29±1.37	0.123	3.08±1.52	3.50±1.45	0.309
Judging and criticizing	2.60±1.50	3.32±1.35	0.064	3.32±1.31	3.73±1.40	0.286
Discounting	2.14±1.24	3.00±1.28	0.023	2.40±1.27	3.26±1.36	0.039
Sexual harassment	2.00±1.36	2.23±1.48	0.646	2.29±1.44	2.63±1.63	0.553
Threatening	2.27±1.55	2.21±1.18	0.864	3.00±1.70	2.79±1.35	0.650

Emotional reactions, cognitive appraisal and coping behaviors and their perceived effectiveness

The emotional reactions subscale describes the victim’s feelings after experiencing verbal abuse (Table 4). The majority of physicians stated that they felt mainly anger (2.94 ± 1.35), disgust (2.58 ± 1.54), and sadness/hurt (2.35 ± 1.37), whereas most of the nurses felt anger (3.49 ± 1.39), disgust (3.05 ± 1.52) and frustration (2.95 ± 1.47). A statistically significant difference was found between physicians and nurses in terms of the emotions they experienced as a victim immediately after verbal abuse. In particular, nurses felt more frustrated (p= 0.011), while physicians felt more indifferent (p = 0.024). The cognitive appraisal subscale describes the cognitive assessment and emotional response/reaction to the incident of verbal abuse (Table 4). The first thoughts mentioned by physicians and nurses were that the abuser had no right (3.09 ± 1.64 and 3.38 ± 1.71), and that they did not deserve to be treated that way (2.91 ± 1.71 and 3.55 ± 1.68) respectively. It is important to be mentioned that nurses were more likely to think that the incident might hurt them, in comparison with the physicians (p = 0.009). The coping behaviors and their perceived effectiveness subscale record how the victim dealt with or managed the episode of verbal abuse (Table 4). Most physicians and nurses responded that they managed the episode by attempting to clarify the possible misunderstanding (2.88 ± 1.27 and 2.53 ± 1.43) and trying to put the situation in perspective (2.71 ± 1.38 and 2.39 ± 1.31). No statistically significant difference was found between physicians and nurses with respect to the management of verbal abuse.

**Table 4:** Emotional reactions, cognitive appraisal and coping behaviors and their perceived effectiveness in physicians and nurses

Emotional reactions, cognitive appraisal, and coping behaviors	Physicians	Nurses	P-value
Emotional Reactions			
Anger	2.94±1.35	3.49±1.39	0.093
Frustration	2.12±1.20	2.95±1.47	0.011
Disgust	2.58±1.54	3.05±1.52	0.193
Embarrassment/humiliation	1.79±1.15	2.00±1.24	0.466
Sadness/hurt	2.35±1.37	2.87±1.58	0.140
Shock/surprise	2.15±1.08	2.62±1.55	0.144
Misunderstood	2.00±1.07	1.90±1.27	0.713
Powerless	1.55±1.02	1.64±1.07	0.738
Helpless	1.55±0.92	1.54±0.94	0.926
Intimidated	1.44±0.86	1.41±0.88	0.880
Overwhelmed	1.71±0.94	1.82±1.14	0.644
Threatened	1.59±0.82	1.79±1.10	0.373
Defeated	1.50±0.83	1.51±0.91	0.950
Shamed	1.91±1.16	1.69±1.15	0.422
Confused	1.97±1.11	1.97±1.25	0.989
Felt responsible	1.70±1.07	1.44±0.68	0.215
Indifferent	2.26±1.40	1.62±0.99	0.024
Fear	1.38±0.74	1.69±1.13	0.176
Cognitive Appraisal			
What a jerk	2.41±1.60	3.03±1.76	0.124
I don't deserve to be treated this way	2.91±1.71	3.55±1.68	0.111
He/she has no right to treat me this way	3.09±1.64	3.38±1.71	0.465
Ι can deal with this	2.79±1.67	2.63±1.55	0.652
I haven't done anything wrong	2.67±1.80	3.15±1.73	0.247
This is horrible, and it could get worse	1.44±0.89	2.03±1.58	0.061
This could potentially hurt me	1.62±0.78	2.35±1.41	0.009
I can't handle this	1.73±1.21	2.25±1.41	0.097
This is no big deal to me	2.12±1.07	1.93±1.23	0.477
I am going to be in trouble over this	1.42±0.90	1.63±1.17	0.422
I must have done something wrong	1.47±0.79	1.63±1.05	0.484
It must be my fault	1.71±0.97	1.80±1.14	0.706
Coping Behaviors and Their Perceived Effectiveness			
I attempt to clarify any misunderstanding	2.88±1.27	2.53±1.43	0.270
I ask for assistance/support from others	1.91±1.11	1.92±1.24	0.974
I try to put the situation in perspective	2.71±1.38	2.39±1.31	0.314
I deal directly with the abuser about the abuse	2.35±1.47	1.95±1.29	0.218
I become silent with the abuser	2.41±1.64	1.89±1.09	0.115
I talk to myself in a nurturing and reassuring way	2.12±1.27	2.29±1.23	0.562
I engage in positive activities that directly reduce my tension	2.71±1.40	2.34±1.19	0.239
I detach myself from the situation	2.38±1.48	2.18±1.20	0.533
I engage in less-than-positive activities that reduce my tension	1.85±0.99	2.00±1.12	0.558
I engage in wishful thinking	1.94±1.07	2.30±1.35	0.225.
I withdraw. I try to keep my feelings to myself and keep others from knowing how bad things are	2.35±1.43	2.53±1.35	0.599
I tend to blame myself	1.29±0.58	1.45±0.76	0.344

Severity of long-term negative effects

Both physicians and nurses stated that after the episode of verbal abuse, the relationships with other health professionals were affected (3.15±2.09, 2.62±1.35 respectively). In addition, for physicians, the sense of well-being at work was limited, whereas for the nurses the job satisfaction was decreased (Table 5).

**Table 5:** Severity of long-term negative effects of abuse in physicians and nurses

Effect of Verbal Abuse	Physicians	Nurses	P value
Confidence in yourself	1.27±0.57	1.51±0.94	0.206
Sense of well-being at work	2.15±1.16	2.21±1.24	0.837
Job satisfaction	1.94±1.01	2.38±1.33	0.188
Your job performance	1.30±0.64	1.56±0.99	0.198
Trust and support at work	2.03±1.11	2.26±1.29	0.428
Relationships with other health professionals	3.15±2.09	2.62±1.35	0.541
Relationships outside work	2.06±1.15	1.51±0.85	0.023
Mental health	1.50±0.71	1.95±1.18	0.059
Physical health	1.65±0.81	1.77±1.01	0.575
Turnover of nursing staff	1.56±0.79	1.38±0.88	0.377
The nursing shortage	1.97±1.02	1.67±1.08	0.228
Patient care outcomes	1.68±0.91	1.54±1.00	0.196
Productivity of work	1.41±0.61	1.59±1.07	0.395

It is important that as an effect of verbal abuse, physicians were more likely to report a change in their relationships outside of work, in comparison to nurses (p=0.023).

## Discussion

This study contributes to the analysis of the phenomenon of verbal abuse in the workplace in health professionals, which is difficult to be measured, standardized, or understοοd. Although verbal abuse is a less extreme form of violence, it is usually more widespread in health care settings compared to physical assault [20,21]. 

Giving that verbal abuse by colleagues or patients is a reality in the health care environment [22,23], the purpose of this study was to investigate the prevalence of verbal violence in the operating room at three general hospitals in the Peloponnese region of Greece.

According to our results, verbal abuse was experienced more frequently by nurses and was more stressful for them, compared to physicians. This may be the case as nurses are the often first and most available personnel throughout the hospital. Their presence in stressful situations such as deaths, waiting to visit a physician, hospital overcrowding, repeated requests by patients and their relatives for special privileges can lead to illogical and tense reactions situations between nurses, other health professionals, and patients. Previous studies agree with the above findings, stating that nurses are most exposed to verbal, emotional, physical, and even sexual abuse [22,23]. On the other hand, physicians enjoy more respect from patients and other colleagues, due to patients’ asymmetric access to information and fear for good care.

While physicians’ abusers were usually their supervisors, for nurses it was usually a physician. Accusing/blaming were noted as the most common forms of verbal abuse, evoking feelings of anger and disgust in the victims, and forcing them to adopt a more dialectical attitude in order to solve the possible misunderstanding, due to the knowledge that such an incidence could seriously affect their relationships with collaborators.

In the present study, physicians stated that they were subjected to verbal violence at least once a year, whereas in nurses these incidents occurred several times during a year. This was similar to the findings of a study conducted in a university hospital in Egypt which showed that verbal violence occurred at least once a month to operating room nurses, and more frequently to head nurses. However, a previous study in a Greek hospital displayed the incidence of verbal abuse in nursing staff several times a week [24-26].

Regarding the working relationship between the victim of verbal abuse and the abuser, approximately three out of 10 physicians declared that the abuser was their supervisor or relatives of patients, while for more than half of the nurses the abuser was another senior member, usually a physician. This finding is in agreement with international studies, where physicians identified to be one of the main sources of verbal abuse in operating rooms [19]. In addition, studies have demonstrated that nurses can be involved as verbal abusers in operating rooms, and also indicated that violence can be produced from various sources [21, 24-27]. On the contrary, other researchers found that the main abusers were patients and their relatives [7,17,27].

Violent verbal behavior was much more stressful for nurses compared to physicians, probably due to the fact that such incidences are more frequent for them. The most common and serious forms of verbal abuse were accusing, blaming and judging, and criticizing, a finding which is in accordance with other studies [7,28]. On the contrary, a study by Manderino and Berkey found that the most frequent and stressful types of verbal abuse encountered by perioperative nurses were abusive, angry behavior [27].

Concerning the emotional reactions after the occurrence of verbal violence, both professional categories in this research were anger and disgust. Numerous international studies have reported that anger is the most common emotional response to verbal abuse and is often associated with negative thoughts and actions [29,30]. Moreover, other researchers found that victims of verbal violence in healthcare settings, felt not only angry but often surprised and overwhelmed [27,30]. Among recipients of abuse, nurses were more easily to be hurt and to feel more frustrated and humiliated than physicians [30]. The first thoughts mentioned by physicians and nurses after an episode of verbal abuse were that the abuser had no right to behave in an inappropriate way and the feelings of low self-esteem. A study by Abdou is consistent with the above finding, showing that the cognitive assessment of health professionals after verbal violence often takes into account self-esteem and self-respect [26].

As far as the management of the episode of verbal abuse, both physicians and nurses attempted to resolve the possible misunderstanding by trying to put the situation in perspective. Other researchers also displayed that nurses choose to cope with verbal violence with a positive attitude, using their higher work experience, and ability to adapt to the special operating room circumstances [28,29].

Finally, nurses and physicians highlighted that the relationships with other colleagues were influenced after the episode of verbal abuse. This finding, similar to other studies, concluded that verbal violence has a negative impact on working conditions affecting patient care, causing absenteeism, reduced efficiency, and resignations [28,30].

Limitations

It is important to mention two main limitations of the study. First, the research was conducted in three hospitals in the Peloponnese region and may not be representative of all hospitals in Greece. Also, despite the fact that the questionnaires were anonymous, participants may not have recorded all possible cases of verbal violence due to fear and insecurity.

## Conclusions

Nurses state that they feel that they are the more frequent victims of verbal abuse than physicians in the operating room, leading them to feel much more stressed and frustrated. The main types of violence experienced by all health professionals were accusing/blaming, which were coped with by employing a dialectical approach. It is important to mention that violence directly affects the relationships of the abused party with other colleagues, having a negative impact on the provision of health care. An important measure of stopping the occurrence of such incidents is the gradual change of organizational culture, the improvement of communication between team members, as well as the implementation of rules and protocols that clarify the rights and obligations of all categories of employees involved in the workplace.
